# Chitosan Coagulation to Improve Microbial and Turbidity Removal by Ceramic Water Filtration for Household Drinking Water Treatment

**DOI:** 10.3390/ijerph13030269

**Published:** 2016-02-27

**Authors:** Lydia S. Abebe, Xinyu Chen, Mark D. Sobsey

**Affiliations:** Department of Environmental Sciences and Engineering, Gillings School of Global Public Health, University of North Carolina, Chapel Hill, Campus Box 7431, Chapel Hill, NC 27599-7431, USA; cxy@live.unc.edu (X.C.); mark_sobsey@unc.edu (M.D.S.)

**Keywords:** water treatment, coagulation-flocculation, chitosan, viruses, bacteria

## Abstract

The use of porous ceramic filters is promoted globally for household water treatment, but these filters are ineffective in removing viruses from water. In order to increase virus removal, we combine a promising natural coagulant, chitosan, as a pretreatment for ceramic water filters (CWFs) and evaluate the performance of this dual barrier water treatment system. Chitosan is a non-toxic and biodegradable organic polymer derived by simple chemical treatments from chitin, a major source of which is the leftover shells of crustacean seafoods, such as shrimp, prawns, crabs, and lobsters. To determine the effectiveness of chitosan, model test water was contaminated with *Escherichia coli* K011 and coliphage MS2 as a model enteric bacterium and virus, respectively. Kaolinite clay was used to model turbidity. Coagulation effectiveness of three types of modified chitosans was determine at various doses ranging from 5 to 30 mg/L, followed by flocculation and sedimentation. The pre-treated supernatant water was then decanted into the CWF for further treatment by filtration. There were appreciable microbial removals by chitosan HCl, acetate, and lactate pretreatment followed by CWF treatment, with mean reductions (95% CI) between 4.7 (±1.56) and 7.5 (±0.02) log_10_ for *Escherichia coli*, and between 2.8 (±0.10) and 4.5 (±1.04) log_10_ for MS2. Turbidity reduction with chitosan treatment and filtration consistently resulted in turbidities < 1 NTU, which meet turbidity standards of the US EPA and guidance by the World Health Organization (WHO). According to WHO health-based microbial removal targets for household water treatment technology, chitosan coagulation achieved health protective targets for both viruses and bacteria. Therefore, the results of this study support the use of chitosan to improve household drinking water filtration processes by increasing virus and bacteria reductions.

## 1. Introduction

According to the United Nations Children’s Fund (UNICEF), approximately 663 million people worldwide lack access to an improved drinking water source [1]. Global efforts to provide access to water and sanitation over the last two decades resulted in decreased child mortality associated with poor water quality and hygiene and inadequate sanitation, declining from 1.5 million deaths in 1990 to approximately 800,000 in 2012 [2]. However, waterborne viruses are still major contributors to the global burden of diarrheal disease morbidity and mortality. According to the Global Enteric Multicenter Study (GEMS) study, a multi-country prospective, case-control study in Africa and Asia, rotavirus, for which there is now a vaccine, is the leading cause of moderate-to-severe diarrhea in infants and children under five [3]. The GEMS study reported that rotavirus increases risk of death 8.5-fold and contributes to growth stunting associated with chronic diarrheal disease [3]. Other waterborne viruses also contribute to morbidity and mortality worldwide, such as noroviruses, the leading global cause of diarrhea, as well as adenoviruses, astroviruses, and hepatitis A and E viruses. Therefore all of these virus must be controlled to provide safe water [4,5,6].

The conventional multi-barrier system to treat drinking water involves chemical coagulation and flocculation, sedimentation, filtration, and disinfection [7]. Coagulation is a critical first step in the removal of microorganisms, turbidity, colloidal natural organic matter (NOM) and some metals [8,9,10]. In fact, water treatment facility surveys have reported coagulation as the most important step (more so than physical filtration) in the physical removal of contaminants from drinking water [11,12]. Research in water treatment practices have increasingly emphasized the need for widely accessible and environmentally sustainable alternatives to the potentially toxic and persistent chemicals currently used. Development of more effective and environmentally friendly coagulants can potentially enhance the reduction of contaminants in subsequent treatment processes, including filtration and chemical or UV disinfection, as well as reduce costs and energy consumption while minimizing persistent and toxic solids residuals.

The physiochemical principle behind coagulation is the reduction of the repulsive electrical potential between typically electronegative colloidal particles in water, such as color, NOM, microorganisms, clays, *etc.*, in such a way that the coagulant causes these suspended, dispersed particles to destabilize and agglomerate to form large, dense structures (flocs) that will precipitate and sediment [13]. The mechanisms by which colloids are removed include a combination of physical-chemical processes, such as charge neutralization, adsorption, formation of complexes with metals, and precipitation [10].

There are a number of different chemical coagulants, such as alum and iron salts, synthetic and natural polymers, and plants based compounds [10]. The most common inorganic coagulant salts used in conventional water treatment are aluminum based, such as aluminum chloride, and ferric based, such as ferric chloride and sulfate [10,14]. However, as summarized in a review by Matilainen *et al.* 2010, adverse consequences can result from the use of inorganic salts in water treatment, including: (1) high volumes of potentially toxic inorganic waste, which requires appropriate disposal; (2) high levels of residuals in treated water, such as aluminum ions and salts, which have been associated with Alzheimer’s disease and other neurological illnesses; and (3) additional chemical requirements for stabilization and corrosion control within distribution systems [10]. The drawbacks of inorganic salts have led to increasing interest in organic based polymers that are biodegradable and non-toxic. 

Promising alternatives to inorganic salts that have gained increased interest are natural coagulants derived from food processing waste because they are biodegradable, low-cost, and non-toxic. Examples of natural coagulants that have been previously investigated include products from *Opuntia* spp. [15] and *Moringa oleifera* [16,17] in addition to acorns and chestnuts [18]. While these plant-based coagulants are sometimes effective for removal of contaminants from water, they have not been successfully employed because they are not widely available in many geographic settings and commercial sources have not been widely developed. However, the widely available biological coagulant chitosan, which has been the subject of research for food and nutrition, cosmetics, drug-delivery, and wastewater treatment, has the potential to be effective in the removal of contaminants from drinking water [19].

Chitosan biopolymers are produced through the de-acetylation of chitin, which is the second most abundant polysaccharide worldwide [19,20]. Chitin can be extracted from fungal species or from the exoskeleton of crustaceans such as crayfish, lobster, prawns, crab and shrimp [19]. Thus, chitin is a naturally available source of biomass that is abundant, nontoxic and biodegradable [19]. Chitin polymers consist of N-acetyl glucosamine (GlcNAc) and glucosamine (GlcN) units, which contain amino (-NH2) groups. The process of de-acetylation influences the amount of the amino groups along the glucosamine structure [20]. When dissolved, the amino groups on the glucosamine units will protonate, resulting in increased solubility and positive charge (cationic property). The positive charge is a critical property for a coagulant, as a coagulant with high positive charge density in water at or near neutral pH, results in efficient removal of negatively charged turbidity and microbes, more so than a coagulant of lower positive charge density or negative charge [20]. Most natural colloids in water, for example, clays, silts, bacteria, viruses, protozoans, *etc.*, carry a negative charge over the pH range typical of natural waters, approximately pH 5–9 [7]. Together, these chemical properties make chitosan a unique and effective biopolymer for inter-particle bridging, aggregation of floc particles and precipitation, and therefore an ideal candidate for use in water treatment [21,22]. Given these favorable attributes, we systematically investigated chitosans derived from chitin for use in removal of microbes from water as a pre-treatment for point-of-use (POU) or community scale water treatment using ceramic filters.

Ceramic water filters (CWFs) are a type POU water purification technology that are effective, both in the laboratory and the field, in improving the microbial quality of water and reducing diarrheal disease in households and communities [23,24,25,26]. CWFs are made of clay, water, and combustible material, such as sawdust, rice husk, or other forms of agricultural waste particles. An additional component in the production of CWFs are silver nanoparticles, which are added either during mixing wet and dry components, or are applied after firing the filter in a kiln. Because of its antimicrobial properties, silver is considered a critical component of the water purification process, as demonstrated in some previous studies [27,28,29,30]. The porous structure of the ceramic media combined with the apparent antimicrobial activity of silver nanoparticles results in two methods of purification: physical removal of microbial contaminants and other particles constituting turbidity, and possibly chemical disinfection of microbes as they passes through the filter and come in contact with silver within the pore spaces. Although CWFs with silver are effective at removal and disinfection of microbes, viruses are smaller than the pore sizes of the CWFs and are not removed efficiently from water. Furthermore, the extent to which silver in filters is capable of inactivating (killing) viruses is uncertain because of conflicting reports in the literature [31].

The use of chemically modified waters soluble chitosans as coagulants was combined with CWFs to evaluate the removal of bacteria, viruses, and turbidity by the combined processes of chitosan coagulation followed by ceramic filtration. Effectiveness was evaluated using chemically defined test water supplemented with kaolinite clay, *Escherichia coli* K011 bacteria and MS2 male-specific (F+) coliphage to model water contaminated with turbidity, bacteria and viruses, respectively. Test water was coagulated using chitosans at varied doses, flocculation was promoted and flocs were precipitated. The pre-treated water (the supernatant water remaining after chitosan coagulation and floc sedimentation) was transferred into the CWF for further treatment by filtration. The reductions of turbidity, bacteria and viruses were compared to the 3 levels of WHO household water treatment (HWT) performance targets, which were developed based on log_10_ reductions linked to health outcomes through epidemiological evidence as being “highly protective”, “protective”, or of “limited protection” [32]. The goal was to enhance the overall removal of viruses, bacteria, and turbidity removal from water by using the proposed dual treatment barriers of coagulation-flocculation-sedimentation followed by microporous ceramic filtration.

## 2. Methods

**Modified Chitosans.** Three types of modified chitosans were evaluated: chitosan hydrochloride (HCl), chitosan acetate (CH_3_COO-), and chitosan lactate (CH_3_CH(OH)CO_2_-) [33]. These were selected based on a previous systematic screen of chemically different chitosans from which the most effective chitosans as coagulants-flocculants for household use were identified, based on reduction of bacteria and virus levels [33]. Chitosan salts were purchased from Heppe Medical Chitosan GmbH.

**CWF Technology.** The ceramic water filters were manufactured locally in Chapel Hill, North Carolina according to the Potters for Peace (PfP) manufacturing process [34]. PfP is the leading non-profit that has established and facilitated ceramic water filter factories worldwide. The clay, grog, and sawdust used to produce the filters were sourced from central North Carolina. The initial mixing and molding of the mixture consisting of clay, sawdust and water were done manually, and the filters were subsequently fired in a fiber-insulated brick kiln according to standard practices and conditions. However, silver nanoparticles were not incorporated into the filters in order to focus on physical removal of viruses, bacteria and turbidity. A lower reservoir consisting of a 5-gallon plastic paint bucket served as a safe water storage unit, and a spigot was attached at the bottom of the bucket to access the filtered water. The flow rates of nine filters were tested by saturating the pores then filling filters with water up to the rim and allowing filtrate water to flow by gravity for 1 h into the lower collection reservoir. Filtrate volume was then measured. The average flow rate was 1.75 L/h and ranged from 1.4 to 2.3 L/h for the nine filters tested. We selected filters with the 6 highest flow rates for the chitosan coagulation and filtration evaluation and used them repeatedly in successive experiments. 

**Overview of evaluation.** Non-pathogenic microbial surrogates for evaluation of bacteria and virus removal were selected based on the WHO household water treatment evaluation list of recommended pathogens and surrogates. Kaolinite clay was used to evaluate turbidity reduction. Filters were sterilized by autoclaving on a wet cycle at 121 °C for five minutes. The lower filtrate collection reservoir was sterilized using 75% ethanol and rinsed using deionized water to remove residual ethanol. Challenge waters were spiked with kaolinite turbidity and test microorganisms at specified target levels, and samples of the untreated water were taken for initial microbial analysis. Chitosan was dissolved and added to the spiked challenge water at specified target concentrations, and 30 min was allowed for coagulation, flocculation and precipitation. After sedimentation the supernatant water was passed through the filter by gravity flow, and filtrate samples (effluent water) were collected post-treatment. These experiments were conducted in triplicates. Microbes in the untreated influent water and the filtrate were analyzed to determine concentrations, and log_10_ microbe reduction values were calculated based on the difference between the log_10_ influent concentration and effluent concentration.

**Challenge Waters.** Test water consisted of phosphate buffered saline and added kaolinite clay to model turbid, natural water. The size of the kaolinite clay was measured using a Zen 3600 Zetasizer Nano, the analysis yielded a mean of 3.21 μm (Z-average, standard deviation of 192 nm). Phosphate buffered saline (PBS), pH 7.5 was prepared by adding the following to 1 L of water according to EPA Method 1623: NaCl; KCl; Na_2_HPO_4_, anhydrous; and KH_2_PO_4_. All chemicals were purchased from Fisher Scientific. The pH of solutions was adjusted using 1 M HCl or NaOH. Kaolinite clay was added to yield a turbidity range of 10 to 15 NTU. The total volume of the challenge water per filter was 3L. Each experiment consisted of three filters and one dose of chitosan. The chitosan doses evaluated, 0, 5, 10, 20 and 30 mg/L, were based on outcomes from initial coagulation-flocculation-sedimentation jar test experiments performed by Soros [33]. The effect of coagulant dosage on the ceramic filter flow rate was not investigated specifically in this study. Although the 3 L volumes of challenge water filtered per test were lower than water volumes typically filtered daily in households, these volumes were considered representative of microbial reduction performance based on separate studies in which incrementally larger sample volumes were filtered per challenge test (unpublished results). Test microbes used were the following: *Escherichia coli* strain K011 (ATCC# 55124) (model bacterium) and male-specific (F+) coliphage MS2 (ATCC# 15597B1) (model enteric virus). Stocks of microbes propagated in the laboratory were diluted and then dosed into test water at an initial concentration of approximately 1 × 10^6^ per L to determine at least 5 log_10_ reductions (99.9999%) by the applied water treatment processes.

**Microbiological Methods.** Bacteria detection and enumeration. The bacterial stock for the challenge experiments was prepared by adding a small quantity of *Escherichia coli* K011 from a frozen stock to tryptic soy broth (TSB) (Difco) and incubating at 37 °C on a shaker set to 100 to 150 rpm overnight (18 to 20 h). Log phase bacteria were prepared by inoculating 50 mL of TSB with 0.5 mL of overnight broth culture and incubating for 1.5 h at 37 °C. Next, the culture was mixed with 40% sterile glycerol in a volume ratio of 1:1, dispensed in 1 mL aliquots and stored at −80 °C. The *Escherichia coli* concentration was 10^9^ colony forming units (CFU) per 1 mL. For challenge experiments, 3 thawed tubes of *Escherichia coli* were added to 3 L of challenge water to give an initial concentration of approximately 1 × 10^6^ cells/mL. *Escherichia coli* strain K011 was enumerated by spreading on 100 × 15 mm Petri plates of Tryptic Soy Agar (TSA) (Difco) supplemented with 50 µG/mL chloramphenicol) at 12–15 mL/plate according to Standard Methods part 9215 C [35]. Water samples of 100 µL volume were spread on plates, which were inverted and incubated for 18 to 24 h at 37 °C. The resulting colonies on each plate were counted.

**Virus propagation and enumeration.** MS2 was propagated in log-phase host *Escherichia coli* Famp (ATCC # 700609) TSB broth cultures containing 15 µG/mL each of streptomycin and ampicillin by incubating at 37 °C on a shaker set to 100 to 150 rpm overnight (18 to 20 h). MS2 was harvested from infected overnight broth cultures by chloroform extraction with 5% chloroform by volume and centrifugation at 2600 g for 30 min at 4 °C. The recovered supernatant virus stock was dispensed in 200–300 μL aliquots and stored at −80 °C. The Single Agar Layer (SAL) assay was used for detection and enumeration of MS2 in water samples according to EPA Method 1602 [36]. Another batch of *Escherichia coli* Famp was grown overnight for log-phase host preparation conducted on experiment days. On the day of the water sample assay, autoclaved, molten 0.5X TSA was tempered to 55–65 °C. Water samples of 100 µL were pipetted onto 100 mm × 15 mm petri dishes for the SAL procedure. Log-phase host *Escherichia coli* cultures were prepared again and optical density was measured to verify adequate growth. Molten agar medium was supplemented with MgCl_2_, streptomycin and ampicillin to achieve concentrations of 0.05 M, 15 µG/mL and 15 µG/mL, respectively. A 4% volume of log phase *Escherichia coli* culture was added to the molten agar medium. This molten agar medium was added in 12–15 mL aliquots to petri dishes containing water samples, swirled to mix, and the agar was allowed to solidify. Petri dishes were covered, inverted and incubated at 37 °C for 18 to 24 h, after which plaques were counted and their numbers recorded.

**Physical-Chemical Parameters.** Turbidity of pre-filtered and post-filtered water was measured as NTU units using a Hach 2100N Turbidity Meter. Percent reduction of turbidity was determined from the difference between the initial and final turbidity, dividing by the initial turbidity, and then multiplying by 100. According to WHO guidelines, turbidity in treated water should not exceed 1 NTU [37]. The pH was analyzed using a Denver Instrument Model 215 meter with combination electrode. 

**Statistical Analysis.** *Escherichia coli* K011 and MS2 concentrations were calculated based on three replicates per dilution. Log_10_ reductions were calculated for influent and effluent waters. All statistical tests were performed using R software. Parametric and nonparametric statistical tests were used to evaluate the difference between microbial reductions when data were normally and non-normally distributed, respectively, as determined by a Shapiro-Wilk normality test (Shapiro and Wilk, 1965). All statistics were interpreted using an a priori defined significance level of α = 0.05. One-way ANOVA and the Friedman tests were used for parametric and nonparametric analysis, respectively, to evaluate the difference between filtration with no pretreatment *versus* filtration with chitosan coagulation pretreatment. If a significant difference existed, paired t-tests were used to perform multiple comparisons between each group of results.

## 3. Results and Discussion

Table 1 presents the log_10_ microbial reductions and water filtrate kaolinite turbidity concentrations reported for samples without chitosan HCl pretreatment and with pretreatments at different chitosan HCl concentrations. Filtration with no pretreatment resulted in a Log_10_ 3.4 (±0.27) reduction of *Escherichia coli*, whereas the use of chitosan HCl pretreatment doses ranging from 5 mg/L to 30 mg/L resulted in average log_10_ reductions ranging from 6.2 to 6.8. All doses of chitosan HCl resulted in significantly (*p* < 0.05) greater reductions of *Escherichia coli* when compared to no pretreatment, but there were no statistically significant differences in log_10_ reductions between different chitosan doses. MS2 log_10_ reduction with no chitosan pretreatment was only 0.10 (±0.11), while with chitosan HCl pretreatment at doses of 5 to 30 mg/L MS2 removal was much greater, ranging from 1.9 to 2.4 log_10_. Pretreatment with chitosan HCl resulted in a significant (*p* < 0.05) reductions of MS2 when compared to filtration only, but there were no statistically significant differences in MS2 log_10_ reductions between different chitosan doses. From an initial turbidity of 9.7 to 13.1 NTU in the untreated test water, final turbidity levels in all filtrate waters ranged from 0.1 to 0.5 and were below the WHO recommended level of 1 NTU. 

Table 2 presents the log_10_ microbial reductions and filtrate water kaolinite turbidity concentrations achieved by different doses of chitosan lactate in test water for pre-treatment coagulation, flocculation and sedimentation, followed by filtration. In these experiments, filtration with no chitosan pretreatment resulted in a log_10_ 4.3 (±0.48) reduction of *Escherichia coli*. The use of chitosan acetate pretreatment doses ranging from 5 mg/L to 30 mg/L resulted in average log_10_ *Escherichia coli* reductions ranging from log_10_ 6.1 to 7.5; this range is more than 2 log_10_ greater than reductions by filtration alone. All doses of chitosan lactate resulted in significant (*p* < 0.05) reductions of *Escherichia coli* when compared to no chitosan pretreatment, but there were no statistically significant differences in log_10_ reductions between the different chitosan doses. MS2 log_10_ reduction with no pretreatment was 0.19 (±0.18). The log_10_ reductions of MS2 with chitosan lactate pretreatment, at doses ranging from 5 to 30 mg/L, followed by ceramic filtration ranged from 3.0 to 3.3 log_10_, which is about a 3 log_10_ increase in MS2 reduction compared to filtration alone. All doses of chitosan lactate resulted in a statistically significant (*p* < 0.05) reduction of MS2 when compared to filtration alone but there were no statistically significance differences in log_10_ reductions between different chitosan doses. From an initial turbidity of 8.7 to 18.7 NTU, final turbidity levels in all filtrate waters were a mean of 0.2 NTU and were below the WHO recommended level of 1 NTU.

Table 3 presents the log_10_ microbial reductions and filtrate kaolinite turbidity concentrations in test water resulting from chitosan acetate pretreatment followed by ceramic filtration. Filtration with no chitosan acetate pretreatment resulted in a mean log_10_ 2.4 (±0.62) *Escherichia coli* reduction. The use of chitosan acetate pretreatment at doses ranging from 5 to 30 mg/L resulted in average log_10_ *Escherichia coli* reductions ranging from 4.7 to 5.4, which is more than 2 log_10_ greater reduction than filtration alone. Only two doses of chitosan acetate (5 and 30 mg/L) resulted in statistically significant (*p* < 0.05) reductions of *Escherichia coli* when compared to no use of coagulant, and there were no statistically significant differences in log_10_ reductions between the different chitosan doses. MS2 log_10_ reduction by filtration with no pretreatment was 0.36 (±0.44) is somewhat higher than the log_10_ reductions reported for experiments with chitosan HCl (0.10 log_10_) and chitosan acetate (0.19 log_10_). However, with chitosan acetate pretreatment at doses of 5 to 30 mg/L followed by filtration, MS2 reduction increased substantially to between 2.8 to 4.5 log_10_. All doses of chitosan acetate resulted in significant increases MS2 log_10_ reductions compared to filtration alone and there were statistically significant differences in log_10_ reductions between the 5 mg/L and 30 mg/L chitosan doses. From an initial turbidity of 8.9 to 11.1 NTU in test water, final turbidity levels in filtered effluent waters were 0.13 to 0.2 NTU, well below the WHO recommended level of 1 NTU. 

## 4. Conclusions

### 4.1. Performance Efficacy

In this study the first results are reported from evaluating the effectiveness of chitosans, as natural polymeric coagulants, combined with subsequent ceramic pot filtration, to reduce the concentrations of bacteria and viruses as well as turbidity in a model drinking water. All three modified chitosans evaluated, chitosan HCl, chitosan lactate, and chitosan acetate, achieved extensive reductions of bacteria, viruses and turbidity to meet performance targets of WHO for HWT technologies and the Guidelines for Drinking-water Quality. The greatest log_10_ reduction of MS2 coliphage, 4.5 (±1.04) was achieved with a dose of 30 mg/L of chitosan acetate and ceramic filtration. However, 2 of the 3 chitosans tested, chitosan acetate and chitosan lactate, achieved the 3 log_10_ reduction “protective” performance level for viruses of the WHO HWT Scheme, and chitosan HCl reduced viruses by up to 2.5 log_10_. By comparison, ceramic filtration without chitosans reduced viruses only by <0.4 log_10_. The greatest log_10_ reduction of *Escherichia coli*, 7.5 (±0.02), was achieved with a 30 mg/L concentration of chitosan lactate and ceramic filtration. Additionally, pretreatment with any of the three chitosans plus ceramic filtration achieved the 6 log_10_ “highly” protective level of bacteria reduction set by the WHO HWT performance scheme. Kaolinite clay turbidity reductions with chitosan pretreatment and filtration consistently produced filtrate water with <0.4 NTU turbidity, well below the 1 NTU level of performance recommended by the WHO GDWQ. However, filtration alone produced low filtrate water turbidity levels of about 0.2 NTU; therefore, pretreatment with any of the chitosans did not further improve the already substantial turbidity reductions of the water by filtration.

A key goal of this study was to evaluate pretreatment of water with chitosan coagulation-flocculation to enhance microbial reductions, and especially virus reductions, by a household water filter. The basis of microbial reduction performance was evaluated according to levels set by the WHO. In 2011, WHO developed performance targets for the evaluation of household water treatment devices based on health risk targets linked to log_10_ microbial reductions. The recommended performance levels consist of a 3-tiered approach for the reduction of bacteria, viruses, and protozoa. The top tier is designated as highly protective with bacteria, virus and protozoan parasite log_10_ reductions of ≥4, ≥5 and ≥4, respectively. The second tier is designated as protective and specifies log_10_ bacteria, virus and protozoan parasite reductions of ≥2, ≥3 and ≥2, respectively. The lowest tier, designated as minimally protective, specifies achieving the “protective” log_10_ reduction levels for two of the three classes of microorganisms. In addition, the lowest performance tier must also provide evidence of health protection, typically from diarrheal disease, based on credible epidemiological field studies.

Based on the WHO performance targets, results of this study demonstrate that the use of chitosan pretreatment prior to ceramic filtration significantly increases the very low 0.1 to 0.36 log_10_ reductions of MS2 virus by filtration only (without chitosan) by another 1.4 to 4.5 log_10_ when using chitosan coagulation-flocculation-sedimentation pretreatment followed by filtration. While the range of tested chitosan HCl doses used did not consistently achieve the 3 log_10_ WHO “protective” level of virus reduction, each dose did increase virus reductions by at least 2 log_10_ , which still provides a considerable reduction in morbidity risks, compared to the very low (<0.4) log_10_ reductions achieved without chitosan pretreatment. Both chitosan acetate and lactate pretreatment followed by filtration achieved or exceeded the “protective” 3 log_10_ reduction target of WHO at multiple coagulant doses, almost achieving a “highly-protective” level of log_10_ reduction performance in the case of the 30 mg/L chitosan acetate dose. These results support the use of chitosan acetate and lactate pretreatment to significantly improve ceramic filtration of household drinking water by reducing virus risks. 

### 4.2. Possible Mechanisms of Microbial Reduction by Chitosans

The mechanisms by which chitosan coagulation-flocculation and sedimentation enhanced microbial removal from water by ceramic filtration were not investigated specifically in this study. Therefore, mechanistic considerations of the physicochemical processes known to be involved are speculative and based only on general principals and plausibility. Evidence from the existing literature suggests that the chitosan polymers studied promote chemical coagulation, subsequent floc formation and sedimentation of viruses and other colloidal particles in the test water through two established processes: interparticle bridging and charge neutralization [19,20]. The predominant parameters thought to be driving coagulation are chitosan molecular weight, the degree of deacetylation, pH and surface electrical potential [19,20]. Chitosans are typically modified by acids such as acetate, lactate and HCl, which dissociate and thereby protonate amino groups present on the biopolymer chain. These amino groups create cationic sites on the chitosan polymers, which facilitate their attraction to and adsorption of negatively charged colloids, such as viruses, bacteria and clay turbidity, thereby promoting their coagulation-flocculation and sedimentation. 

### 4.3. Performance Compared to Prior Studies of Natural Polymer Coagulation of Water

The majority of previous studies on the use of natural coagulant polymers to treat water investigated primarily turbidity and bacteria reductions. A study by Babu and Chaudhari (2005) investigated the direct filtration of water after coagulation with material from Strychnos potatorum and *Moringa oleifera* seeds and reported reductions of turbidity, heterotrophic bacteria, and fecal coliforms [17]. Another study on coagulation by *Moringa oleifera* material concluded that the resulting residual solids (sludge) were not harmful to the environment and the mass produced was four to five fold less than alum sludge residual [16]. Miller *et al.* (2008) evaluated a polymer extract of the cactus *Opuntia* spp. for coagulation of waters with a wide range of initial turbidity levels and reported 98% turbidity reductions [15]. Sciban *et al.* (2003) used material from chestnuts and acorns for coagulation and reported approximately 80% and 70% turbidity reductions, respectively [18]. However, despite increasing interest in the use of such natural coagulants for water treatment, most studies report only turbidity reductions, a few have investigated bacteria reductions, none have investigated virus reductions and none have employed them as a pretreatment prior to filtration. In order to address the limitations of previous studies, this study focused on the use of chitosan coagulants as a pretreatment for household water filtration to improve virus removal. The combination of pretreatment with chitosan natural coagulants followed by flocculation, sedimentation and then filtration with ceramic pot filters (CWFs) greatly increased virus reductions from water and also provided effective reductions of bacteria and turbidity. 

### 4.4. Limitations and Future Research

Results from this study demonstrate that doses of chitosan salts enhance significantly the removals of model microorganisms from water. While this research is a first effort to demonstrate enhanced microbial reductions by chitosan coagulation followed by microporous filtration from a model water, the possibilities of unintended effects such as greater filter clogging or increases in pH were not evaluated systematically in this study. However, addition of these doses of chitosan salts to a buffered test water did not appreciably change pH in previous coagulation-flocculation-sedimentation studies [33]. There are limitations in extending these results to field use conditions because the challenge water used for these experiments was a chemically defined model water seeded with model microorganisms, and an inorganic clay, kaolinite, as the source of turbidity. Further studies are needed with other water qualities having varied sources of turbidity and natural organic matter and additional representative microorganisms before extending these findings to in home use. However, to our knowledge, this is the first report documenting the ability of polymeric chitosan salts to greatly enhance virus removals as well as further improve bacteria removal while maintaining turbidity removals from water when used as a dual treatment barrier consisting of coagulation-flocculation-sedimentation followed by microporous ceramic filtration. Further studies are recommended to evaluate the reductions of other viruses as well as other bacteria, protozoans and turbidity types from waters of different quality and to evaluate this dual barrier chitosan coagulation and filtration system under field conditions. 

## Figures and Tables

**Table 1:** Log_10_ Microbial Reductions and Final Effluent Turbidity of Test Water by Chitosan HCl Coagulation with and without Ceramic Filtration. Mean (±SD) for 3 Replicate Experiments per Condition.

Chitosan Concentration and Filtration Status	*Escherichia coli* K011 Log_10_ Reduction	MS2 Log_10_ Reduction	Effluent Turbidity (NTU)	Coagulation Pretreatment + Filter *vs.* Filter Only	
				*Escherichia coli* K011	MS2
Filtration (F) Only (No Chitosan HCl)	3.4 (±0.27)	0.1 (±0.10)	0.2 (±0.06)	--	--
5 mg/L+ F *	6.2 (±1.03)	1.4 (±0.30)	0.5 (±0.17)	**S	S
10 mg/L + F	6.4 (±0.05)	2.4 (±0.26)	0.3 (±0.08)	S	S
20 mg/L + F	6.7 (±0.09)	2.0 (±0.31)	0.1 (±0.03)	S	S
30 mg/L + F	6.8 (±0.03)	1.9 (±0.29)	0.1 (±0.04)	S	S

SD: Standard Deviation; * Different baseline; **S = Statistically significant difference.

**Table 2:** Log_10_ Microbial Reductions and Final Effluent Turbidity of Test Water by Chitosan Lactate Coagulation with and without Ceramic Filtration. Mean (±SD) for 3 Replicate Experiments per Condition.

Chitosan Concentration and Filtration Status	*Escherichia coli* K011 Log_10_ Reduction	MS2 Log_10_ Reduction	Effluent Turbidity (NTU)	Coagulation Pretreatment + Filter *vs.* Filter Only	
				*Escherichia coli* K011	MS2
Filtration (F) Only (No Chitosan Lactate)	4.3 (±0.54)	0.2 (±0.18)	0.2 (±0.00)	--	--
5 mg/L + F	6.1 (±0.30)	3.1 (±0.4)	0.2 (±0.00)	S	S
10 mg/L + F	6.3 (±0.32)	3.0 (±0.25)	0.2 (±0.00)	S	S
20 mg/L + F	6.4 (±0.06)	3.2 (±0.28)	0.2 (±0.00)	S	S
30 mg/L + F	7.5 (±0.02)	3.2 (±0.40)	0.2 (±0.07)	S	S

**Table 3:** Log_10_ Microbial Reductions and Final Effluent Turbidity of Test Water by Chitosan Acetate Coagulation with and without Ceramic Filtration. Mean (±SD) for 3 Replicate Experiments per Condition.

Chitosan Concentration and Filtration Status	*Escherichia coli* K011 Log_10_ Reduction	MS2 Log_10_ Reduction	Effluent Turbidity (NTU)	Coagulation Pretreatment + Filter *vs.* Filter Only	
				*Escherichia coli* K011	MS2
Filtration (F) Only (No Chitosan Lactate)	2.4 (±0.62)	0.4 (±0.40)	0.2 (±0.00)	--	--
5 mg/L + F	5.4 (±0.82)	2.8 (±0.10)	0.2 (±0.00)	S	S
10 mg/L + F	4.9 (±1.12)	3.3 (±0.21)	0.2 (±0.00)	*NS (*p* > 0.05)	S
20 mg/L + F	4.7 (±1.56)	3.5 (± 0.52)	0.2 (±0.10)	NS (*p* > 0.05)	S
30 mg/L + F	5.4 (±1.31)	4.5 (± 1.04)	0.1 (±0.06)	S	S

*NS = No statistically significant difference.
